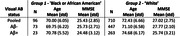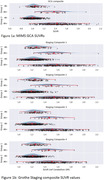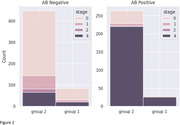# Comparison of local amyloid PET uptake patterns between black and white participants in the GAP Bio‐Hermes study

**DOI:** 10.1002/alz.092157

**Published:** 2025-01-09

**Authors:** Robin Wolz, Colm J McGinnity, Richard Joules, Lynne Hughes, Richard Mohs, John Dwyer, Douglas W. Beauregard

**Affiliations:** ^1^ IXICO, London UK; ^2^ Global Alzheimer's Platform Foundation, Washington, DC USA

## Abstract

**Background:**

Amyloid status is a core marker of Alzheimer’s disease and can be defined qualitatively through expert neuroradiologist visual read and quantitively through SUVR analysis. We investigate differences in amyloid status classification between racial groups.

**Method:**

We analysed data from 944 participants enrolled in the Global Alzheimer’s Platform (GAP) Bio‐Hermes trial. After exclusion of technical failures (N=9), subgroups in the ‘Non‐Hispanic or Latino’ population were defined as follows: Group 1 ('Black or African American') and Group 2 (‘White’; see Table 1).

**Result:**

GCA SUVR was not statistically different between Groups 1/2 for pooled data. Figure 1a reveals that Aβ‐ participants in Group 1 had notably less occurrence of lower GCA SUVR values compared with Group 2, which resulted in a higher mean SUVR, driving the significant group difference observed between Aβ‐ participants (p<0.001).

Aβ+ fraction based on visual read was 24% in Group 1 and 37% in Group 2; these fractions change to 38% and 41% respectively with a threshold of GCA SUVR > 1.1.

Significant group differences were observed for Aβ‐ participants in the SUVRs for Grothe Stages 2, 3, and 4 (p <0.001, Figure 1b).

In Aβ‐ subjects, statistically significantly higher SUVR was observed in ‘Black or African American’ participants (Group 1) compared to ‘White’ participants (Group 2) for the caudate, medial temporal, parietal and frontal lobes (p < 0.05 with Bonferroni correction).

**Conclusion:**

Our findings suggest there may be differences in patterns of amyloid deposition between racial groups that may confound quantitative and visual classification of amyloid status. Further study is required to understand a potential impact on determining amyloid state for populations under‐represented in the definition of current criteria.